# Towards a Complete DNA Barcode Library of Austrian Lepidoptera

**DOI:** 10.3390/insects17050473

**Published:** 2026-05-03

**Authors:** Peter Huemer, Wolfgang Stark, Christian Wieser, Peter Buchner, Johannes Rüdisser, Paul D. N. Hebert, Benjamin Schattanek-Wiesmair

**Affiliations:** 1 Naturwissenschaftliche Sammlungen, Sammlungs- und Forschungszentrum, Tiroler Landesmuseen Betriebsges.m.b.H., Krajnc-Str. 1, 6060 Hall in Tirol, Austria; b.wiesmair@tiroler-landesmuseen.at; 2 Department of Ecology, University of Innsbruck, 6020 Innsbruck, Austria; johannes.ruedisser@uibk.ac.at; 3 Ökoplus Umweltforschung und Consulting GmbH, Stockerauer Str. 16, 3430 Trübensee, Austria; wolfgang.stark@oekoplus.co.at; 4 Independent Researcher, Sternengasse 13, 9064 Magdalensberg, Austria; wieser.ch@aon.at; 5 Natural History Museum Vienna, Burgring 7, 1010 Wien, Austria; buchner.324@drei.at; 6 Centre for Biodiversity Genomics, University of Guelph, 150 Stone Road East, Guelph, ON N1G 2W1, Canada; phebert@uoguelph.ca

**Keywords:** Alps, cryptic diversity, Europe, faunistics, mt DNA, taxonomy

## Abstract

This study presents the first comprehensive molecular assessment of the Lepidoptera fauna of Austria based on DNA barcodes (cytochrome *c* oxidase I, COI; 658 bp Folmer region). The barcode reference library comprises approximately 23,500 sequences, representing 3591 Linnaean species or about 85% of the known national species (ca. 4200 species). Congruence between morphological species identifications under the Linnaean system and barcode data was evaluated using the Barcode Index Number (BIN) system. A total of 244 species could not be unambiguously assigned, showing two to seven BINs that exhibit elevated genetic divergence and may partially represent cryptic diversity. These taxa, together with 40 currently unnamed lineages, require further integrative taxonomic assessment. The distinctiveness of the Austrian Lepidoptera fauna is discussed in the context of endemic genetic diversity. Finally, 17 new faunistic records for Austria are reported.

## Simple Summary

Although DNA barcoding is an important tool for identifying species and studying biodiversity, comprehensive DNA barcode libraries for insects are still rare in Europe. Our study addresses this gap by providing a nearly complete reference library for the butterflies and moths (Lepidoptera) of Austria. Using DNA barcoding, we built a reference library with about 23,500 sequences, covering around 85% of all known species in the country. By comparing traditional species identification with genetic data, we found that most species can be reliably identified using DNA barcodes. However, nearly 7% of the species showed high intraspecific differences, indicating potential cryptic diversity. In addition, several previously unrecognized genetic lineages were detected and require further study. Overall, our results improve the understanding of Austria’s Lepidoptera, highlight the importance of expanding DNA barcode libraries and combining molecular and traditional methods to better document and protect biodiversity.

## 1. Introduction

Austria is a country of striking landscape and cultural diversity located in central Europe. Covering an area of 83,884 km^2^, it has a population of about 9.2 million inhabitants. Administratively, the country is divided into nine federal states.

Austria comprises three major biogeographical regions: The Alps cover 63% of the country’s area and culminate at Großglockner (3798 m a.s.l.), the highest peak in Austria. The continental region encompasses northern and northeastern Austria, specifically the Northern Alpine Foreland and the Danube Basin. The Pannonian Region in the south east represents a warm, dry steppe-influenced environment that shares species with the Hungarian Plain. Within this region lies the lowest point of Austria at 113 m above sea level [1]. Austria has a mostly temperate climate, ranging from oceanic to continental influence. However, there are clear regional differences due to the country’s varied landscape and the increasingly apparent impact of climate change [1]. Yearly precipitation varies between 500 and 2500 mm, while the average annual temperature ranges from about 10 °C down to below −5 °C at the highest elevations [2]. Western Austria is influenced predominantly by oceanic air masses, resulting in relatively humid conditions with cool summers and comparatively mild winters. In southern regions, Mediterranean circulation patterns frequently influence weather conditions. In contrast, eastern Austria exhibits a Pannonian-continental climate, characterized by hot summers, cold winters, and comparatively low precipitation. At regional scales, climatic conditions are strongly modified by Alpine topography, resulting in climatic gradients ranging from montane to nival environments. In addition, the main Alpine ridge acts as a major climatic divide and strongly influences local weather patterns.

**Figure 1:**
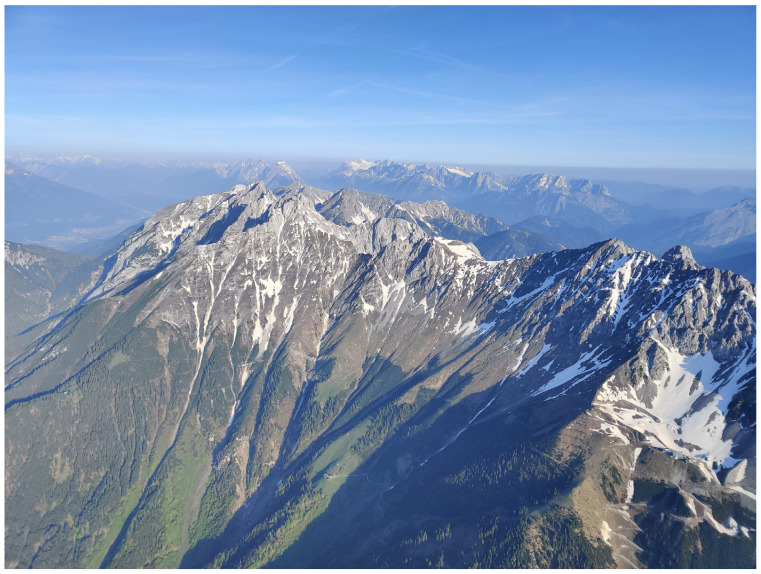
Large parts of Austria are dominated by the Alps (North Tyrol) (Photo P. Huemer).

**Figure 2:**
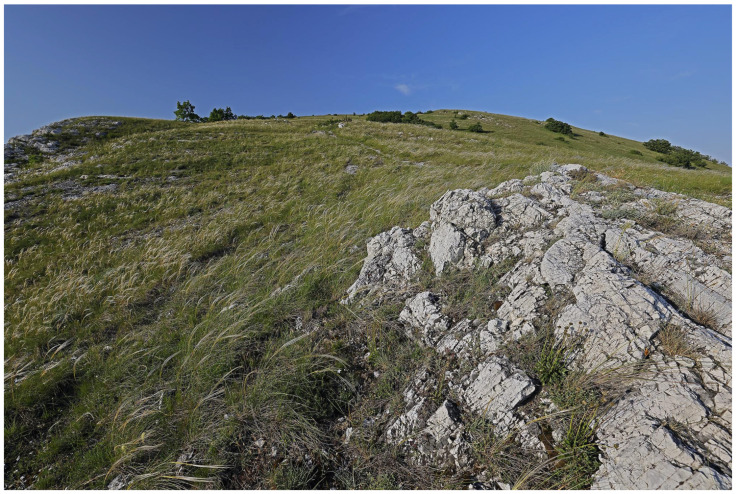
Steppe slopes in the Hundsheimer Berge (Lower Austria). Reproduced with permission from G. Rotheneder.

**Figure 3:**
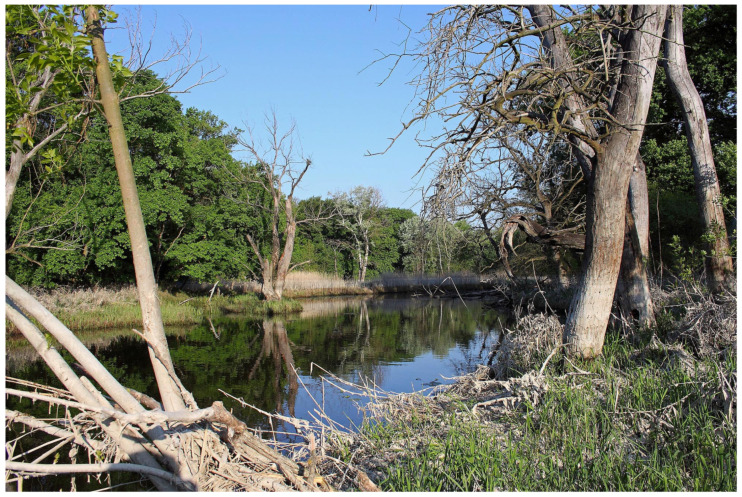
Lowland floodplain forest along the March River (Lower Austria). Reproduced with permission from G. Rotheneder.

**Figure 4:**
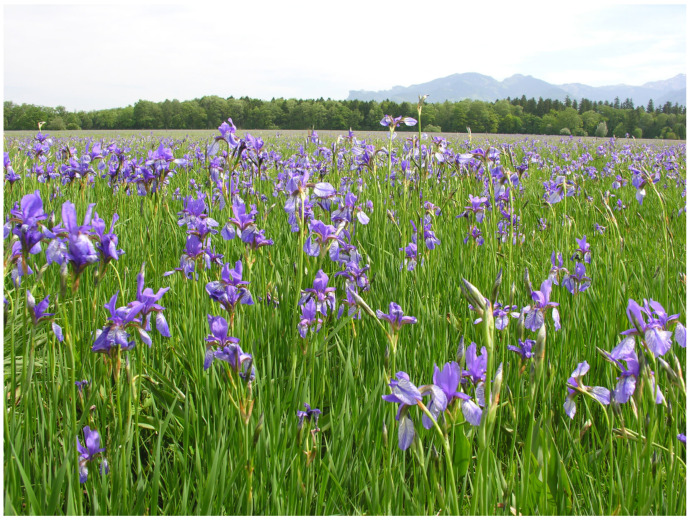
Extensive meadows with a large population of Iris sibirica in the Rhine Valley (Vorarlberg) (Photo P. Huemer).

**Figure 5:**
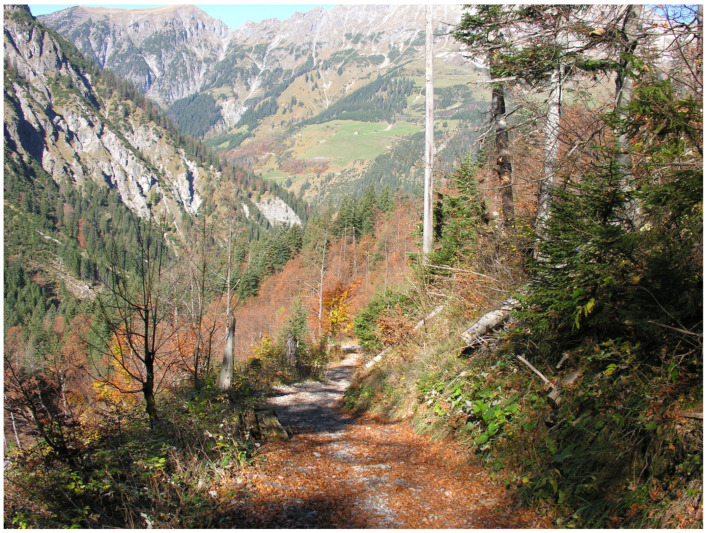
Mixed coniferous–deciduous forests cover nearly half of the country’s area; shown here is the Gadental (Vorarlberg) (Photo P. Huemer).

**Figure 6:**
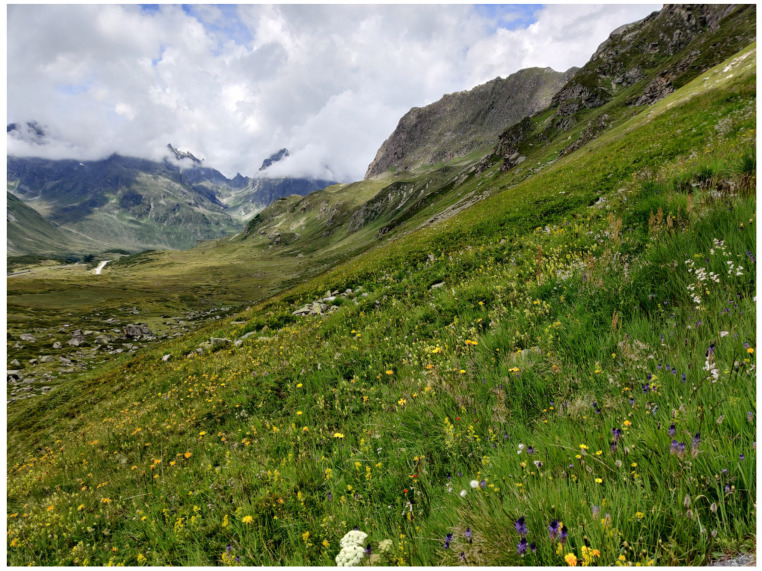
Flower-rich mountain hay meadows in western Austria (Vorarlberg) (Photo P. Huemer).

**Figure 7:**
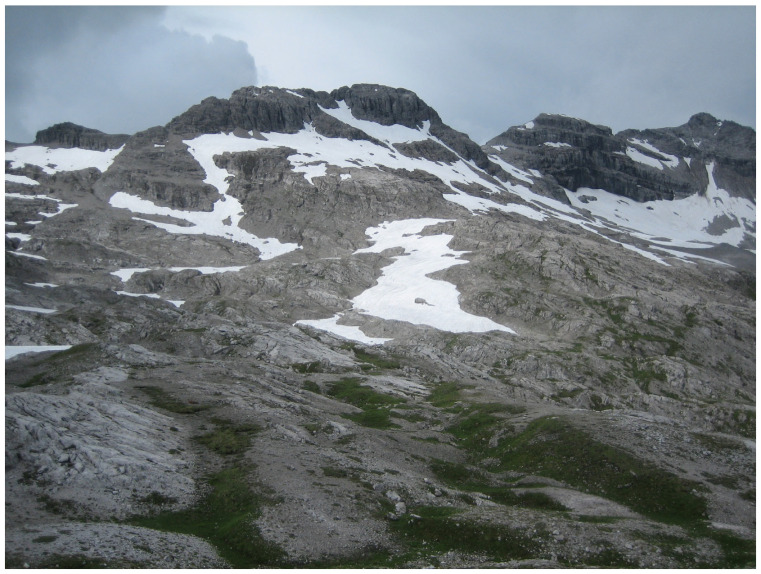
Sparsely vegetated rock and scree fields in the subnival zone (Vorarlberg) (Photo P. Huemer).

The resulting environmental heterogeneity supports an exceptionally high diversity of habitats (Figure 1, Figure 2, Figure 3, Figure 4, Figure 5, Figure 6 and Figure 7). In total, nearly 500 different biotope types have been documented within Austria [3]. Forest habitats and aquatic ecosystems in particular exhibit remarkable diversity, whereas this diversity is less pronounced in grasslands. Of the 383 biotope types considered valuable from a nature conservation perspective, about three quarters are threatened to varying degrees [3]. This threat is also reflected in different animal groups, with nearly 50% of the assessed butterfly and moth species groups classified as threatened [4].

With more than 4200 species of Lepidoptera, Austria is nevertheless considered one of the hotspots for this insect group in Europe. The systematic study of the national fauna has a long tradition, not least because of this remarkable diversity. More than 250 years ago, the Jesuits Michael Denis (1729–1800) and Ignaz Schiffermüller (1727–1806) documented the butterflies and moths of the Vienna region in the monumental work “Ankündung eines systematischen Werkes von den Schmetterlingen der Wienergegend” [5]. In this outstanding regional faunal survey of Lepidoptera based on the then newly introduced Linnaean classification system, hundreds of species were described and named for the first time. Lepiforum https://lepiforum.org/ (accessed on 7 April 2026) lists no fewer than 481 taxa, including several famous species such as Europe’s largest moth, *Saturnia pyri* [6]. Most of the names introduced at that time remain valid today. Famous Austrian contemporaries such as Nikolaus Poda von Neuhaus (1723–1798), Giovanni Antonio Scopoli (1723–1788), and Franz von Paula Schrank (1747–1835) dealt only marginally with butterflies and moths, or their works focused on regions that are no longer part of Austria today.

Until the second half of the nineteenth century, Austrian Lepidoptera received relatively little attention in major entomological works, although Austrian species were included in important foundational publications on the European fauna, particularly those by Jakob Hübner (1761–1826), Ferdinand Ochsenheimer (1767–1822), Friedrich Treitschke (1776–1842), and Gottlieb August Herrich-Schäffer (1799–1874) [7,8,9,10]. One of the early contributions to Austrian lepidopterology during this period, distinguished by the remarkable detail of both text and illustrations, was published by Josef Emanuel Fischer von Röslerstamm (1787–1866) [11].

From the late 19th century and throughout the 20th century in particular, numerous researchers—mostly enthusiastic amateur lepidopterists and occasionally scientists from academic institutions—contributed to the current knowledge of Austria’s Lepidoptera fauna. Among the well-known experts are Ernst Arenberger (1933–2020), Karl Burmann (1908–1995), Franz Gradl (1876–1954), Heinz Habeler (1933–2017), Fritz Hoffmann (1873–1945), Josef Klimesch (1902–1997), Rudolf Klos (1859–1919), Karl Kusdas (1899–1974), Ernst Rudolf Reichl (1926–1999), Hans Reisser (1896–1976), Otto Sterzl (1910–1969), and Josef Thurner (1889–1975).

Professional lepidopterists, however, were always rare exceptions. Among those who made particularly significant contributions to the study of the fauna of the former Austro-Hungarian Monarchy were Alois Rogenhofer (1831–1897), Hans Rebel (1861–1940), and Friedrich Kasy (1920–1990), who served as scientific curators at the Natural History Museum in Vienna, as well as Josef Mann (1804–1889), who worked there as a preparator and collector. Not least because the Republic of Austria assumed its present territorial boundaries only after the end of the First World War, it took until relatively recently for a first comprehensive overview of the national fauna to be published [12,13]. Since then, about 200 additional species have been recorded, bringing the current checklist to ca. 4200 species [6].

For more than 200 years, the faunistic and taxonomic study of Austrian Lepidoptera was based exclusively on morphological criteria. Only in recent years have genetic data increasingly been incorporated, particularly DNA barcodes. Based on approximately 15 years of research, within the framework of regional projects and the National Austrian Barcode of Life (ABOL) initiative some important findings have been published in separate papers. As a result, a largely complete barcode library is available for three species-rich superfamilies: Papilionoidea, Geometroidea, and Noctuoidea. However, these groups comprise only about a third of Austria’s fauna [14,15,16], meaning that comprehensive analysis of Austria’s DNA barcode library has not been available, a gap addressed by this paper.

## 2. Materials and Methods

### 2.1. Sampling and Material

#### 2.1.1. Taxonomic Coverage–Species Inventory

This genetic inventory of the butterflies and moths in Austria was based on the systematic collection of representatives of as many species as possible. A taxonomically comprehensive sampling plan for the establishment of a national DNA barcode library was derived from the Austrian checklist [13]. This inventory was subsequently incorporated into Lepiforum, where it is maintained exclusively online and regularly updated to reflect the latest faunistic and taxonomic developments [6]. In total, this checklist includes migrants, non-established and established alien species, as well as new records published herein, comprising ca. 4200 species. The present study is based on this faunistic directory and follows the taxonomy, nomenclature, and systematics adopted in Lepiforum. However, taxa that can currently be identified only to the genus level based on genetic and morphological evidence are not considered in the Lepiforum checklist.

#### 2.1.2. Geographic Coverage–Sampling Plan

This study encompasses DNA barcode sequences of all Lepidoptera specimens from Austria that were accessible to us, including material from various external sources available in BOLD, particularly from the Zoological State Collection (Munich, Germany), the University of Oulu (Oulu, Finland), and the Naturalis Biodiversity Center (Amsterdam, The Netherlands). However, it primarily relies on material collected by several of the authors, particularly Peter Huemer, and Benjamin Schattanek-Wiesmair (TLMF, Tyrolean Federal State Museums, Innsbruck, Austria), as well as Wolfgang Stark, Peter Buchner and Christian Wieser (kärntenmuseum, Klagenfurt, Austria). The sampled material originates exclusively from Austrian territory. The inclusion of otherwise missing species from neighboring countries was deliberately avoided.

To ensure the most comprehensive possible survey of species diversity while also capturing potential geographically driven barcode variability, Austria was divided into three major regions that correspond to political provinces and partly reflect biogeographic areas: (a) northeastern Austria (Burgenland, Vienna, Lower Austria, Upper Austria), (b) southern Austria (Styria, Carinthia, East Tyrol), and (c) western Austria (Salzburg, North Tyrol, Vorarlberg).

A total of 25,642 specimens were analyzed. For the already published superfamilies, Papilionoidea, Geometroidea and Noctuoidea, a minimum of four specimens covering all three regions was targeted wherever feasible [14,15,16]. However, these requirements could not be met for the entire species inventory, as many species are difficult to collect. The primary objective, nevertheless, was to obtain at least one sequence per species wherever possible.

#### 2.1.3. Validation of Identifications

Initial species identification was based on phenotypic and/or genitalia characters. The identifications were subsequently verified using DNA barcodes by constructing a neighbour-joining (NJ) tree in MEGA12 under the Kimura 2-parameter (K2P) model of nucleotide substitution [17], including only sequences longer than 400 bp. Suspected misidentifications were checked against specimen photographs, and records in which the photograph and the genetic sequence did not correspond were removed from the dataset. Furthermore, all species were examined for BIN sharing, and the accuracy of these identifications was verified using morphological characters. Possible intra-BIN barcode divergences of these species were examined using a Neighbor-Joining tree that included all BIN members.

### 2.2. DNA Barcoding

Within the framework of the project, tissue samples (dried legs) from 25,642 specimens, provisionally identified to the morphospecies level, were prepared according to standardized protocols in order to obtain DNA barcode sequences of the mitochondrial COI gene (cytochrome *c* oxidase subunit I). Most of the material was processed at the former Canadian Centre for DNA Barcoding (CCDB, Centre for Biodiversity Genomics, University of Guelph, Guelph, ON, Canada), now the Centre for Biodiversity Genomics, using a standard high-throughput protocol [18]. However, some sequences were generated in other laboratories (see dataset). Complete voucher data and images for the finally successfully sequenced and analyzed 23,463 specimens with sequences longer than 400 bp are available in the public dataset [DS-BCAUT], “Barcodes of Austrian Lepidoptera,” https://doi.org/10.5883/DS-BCAUT (accessed on 7 April 2026) in BOLD [19,20]. In addition to the large majority of barcodes generated in this study or published earlier [14,15,16], the dataset comprises all available sequences in BOLD, particularly those from the Zoological State Collection Munich (Germany), the University of Oulu (Finland), and the Naturalis Biodiversity Center, Amsterdam (The Netherlands), as well as a limited number of publicly available sequences from other institutions and private individuals. Additionally, a complete mitochondrial genome of *Erebia stirius* from Austria (GenBank accession number OZ182028.1) was considered in the analysis.

All sequences were assigned to Barcode Index Numbers (BINs), algorithm-based operational taxonomic units that provide an accurate proxy for species [21]. BINs were automatically calculated for records in BOLD that comply with the DNA Barcode standard. Some BINs included specimens belonging to more than one taxon because of BIN sharing, whereas initial misidentifications or contaminations were corrected and/or deleted.

To visualize the geographical distribution of the BINs, we created a map showing the number of BINs per sampling site. To improve visualization and account for sampling sites that were very close together, we aggregated sites within hexagons with a diameter of 5 km.

BINs restricted to Austria were identified through a database query in the Barcode of Life Data System conducted by the BOLD online support team to retrieve BINs represented exclusively by Austrian records.

Levels of intra- and interspecific variation in the DNA barcode region were calculated under the Kimura 2-parameter model of nucleotide substitution using the analytical tools of BOLD Systems v. 4.0 [20].

## 3. Results

### 3.1. Taxon Coverage

**Figure 8:**
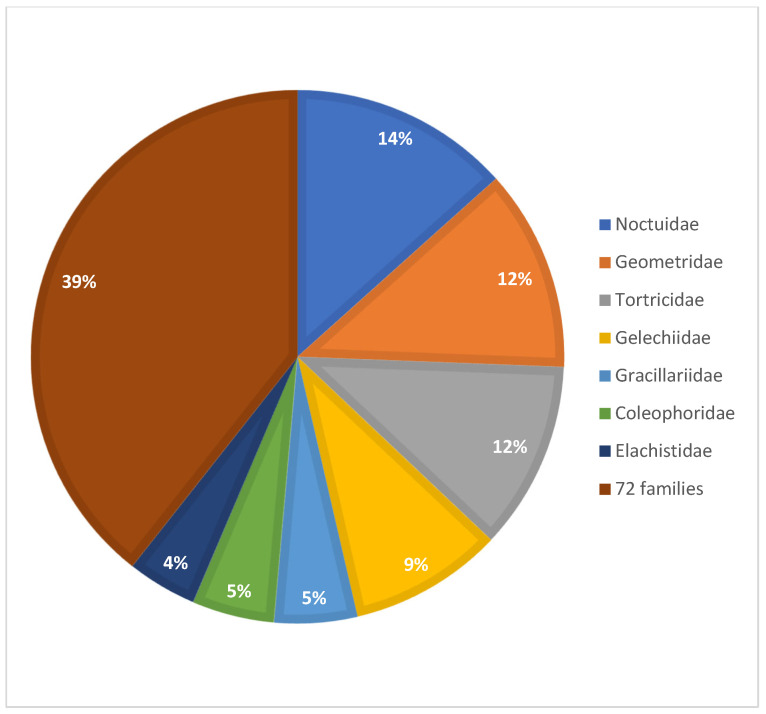
Proportion of DNA barcode sequences per family.

23,463 DNA barcode sequences >400 bp are available for representatives of 79 families of Austrian Lepidoptera, including 23,429 sequences that could be assigned to a BIN. By far the highest numbers of sequences were obtained for the following superfamily and families: Noctuoidea (3136), Geometridae (2873), Tortricidae (2685), and Gelechiidae (2190) (Figure 8). Only four families, each represented by a single species (Bombycidae, Castniidae, Heliodinidae, and Pterolonchidae), have no sequences.

**Figure 9:**
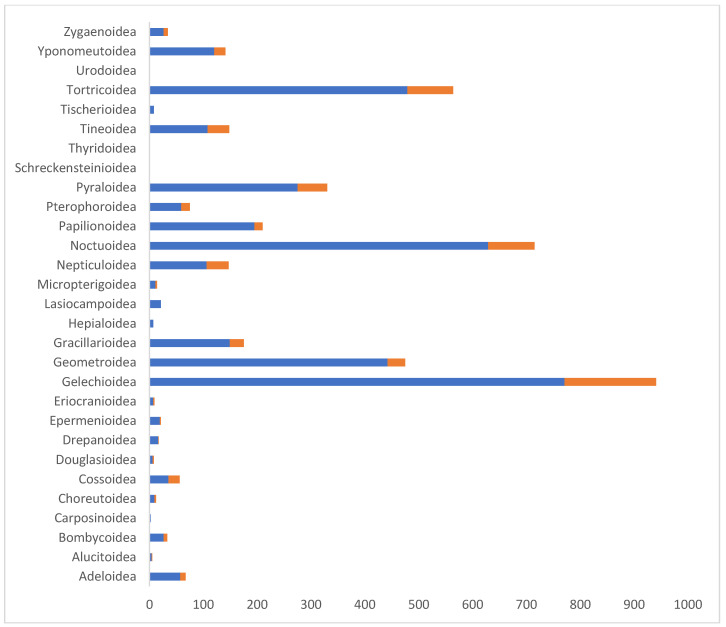
Number of Linnaean species per superfamily in Austria (alphabetic order), with barcode sequence > 400 bp (blue) and without barcode sequence (orange).

Overall, the coverage of barcoded species is extraordinarily high, with 3591 species, or 84.7% of the inventory, out of a total of 4238 Linnean species (Figure 9). The coverage of the sequenced species inventory exceeds 70% in all superfamilies except Cossoidea but only reaches completeness in four species-poor superfamilies. Particularly species-rich superfamilies of the Macrolepidoptera, such as Noctuoidea and Geometroidea, are covered at just under or over 90%, with even the highly diverse Gelechioidea achieving more than 80% coverage.

Among the 647 species lacking records, most are extremely rare taxa, often only known from historical records. In addition, there are a further 40 successfully sequenced taxa that are currently not identifiable to the species level (Table S1).

### 3.2. BIN-Based Species Identification: Potential and Limitations

#### 3.2.1. Linnaean Species with Specific BINs

3341 Linnaean species within the Austrian fauna can be unambiguously identified using the BIN system and most of them (3021) are represented by a single BIN. Approximately 10% of the sequenced species show variability in their DNA barcodes and are assigned to between two and a maximum of seven BINs. The vast majority of these species (277), however, cluster into only two BINs, whereas ten species are assigned to at least four BINs.

Potential cryptic diversity appears possible in several cases of such intraspecific barcode variation and requires further integrative taxonomic analyses, as, for example, in the Gelechiidae *Acompisa maculosella*, *A. tripunctella* and *Oxypteryx libertinella* or in *Mompha miscella* (Momphidae), whereas it has already been ruled out for other species such as *Hepialus humuli* [22,23].

**Figure 10:**
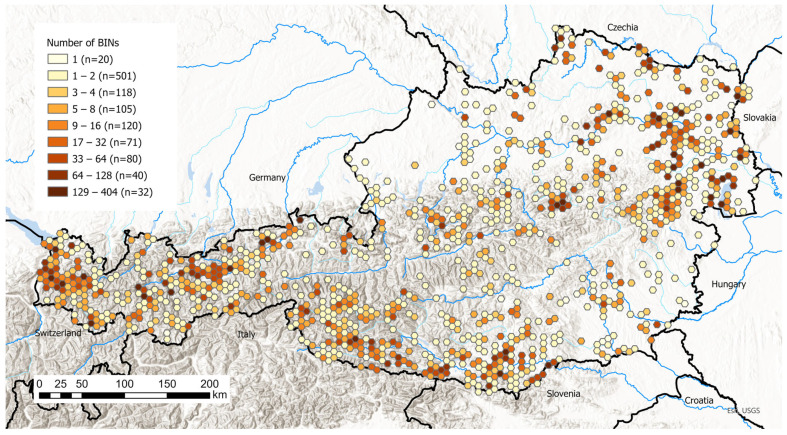
Number of identified BINs per sampling site. Sampling sites that were close together were aggregated within hexagons with a diameter of 5 km. Map data source: ESRI, USGS, UBA.

BINs were aggregated into 1087 hexagons. The number of unique BINs per 5 km hexagon ranged from 1 to 404, with a median of 3, and showed pronounced geographic bias due to differences in sampling intensity (Figure 10).

#### 3.2.2. Species Lacking Distinctive BINs

A total of 244 Linnaean species (104 BINs), corresponding to approximately 6.7% of the sequenced species, cannot be identified to the species level based on their BIN even within Austria as they share their BIN with at least one other species sequenced in the region. Numerous cases of BIN and barcode sharing, as well as overlap, have already been documented, and various underlying factors—such as widespread introgression, often occurring in phylogenetically young species complexes—have been discussed [14,15,16,24,25,26,27]. In other cases, BIN sharing may result from insufficiently justified species concepts and the associated oversplitting or from potential misidentification, as observed, for example, in the family Psychidae.

BIN sharing was detected in 28 families and occurs most frequently in the following groups: Coleophoridae (16 spp.), Depressariidae (17 spp.), Geometridae (19 spp.), Noctuidae (28 spp.), Nymphalidae (18 spp.), and Tortricidae (52 spp.). However, barcode sequences from several of these species form distinct clusters, so the species can therefore be differentiated based on their sequences (Table S2). Particularly in the case of only minimally divergent clusters, a sufficiently large number of samples is essential. Based on the more comprehensive sampling now available in BOLD, in contrast to earlier studies, no reliable genetic distances have been identified for several pairs of species, such as *Photedes captiuncula*/*P. minima* [24]. Moreover, the presumed genetic distances are unreliable in some cases when additional samples are analyzed on a supra-regional scale, for example in *Erebia euryale*/*E. ligea* and *Polyommatus argyrognomon*/*P. idas*. An interesting example is *Colias palaeno*, which shares the BIN BOLD:AAA3447 with *C. chrysotheme* and *C. phicomone* and cannot be distinguished based on sequence data; however, it also occurs in a second BIN (BOLD:AAA4771), which is also present in a North American species.

Overall, morphological examination appears to be essential for reliable species delimitation in all these species. Nevertheless, there is a large number of species that can be readily distinguished based on phenotype and genital morphology.

#### 3.2.3. Taxonomically Unresolved BINs—Potential Cryptic Diversity

Forty species sequenced in our study could not be assigned to a Linnaean species based on their BIN, despite initial morphological examination (Table S3). Thirty-seven species belong to particularly difficult and taxonomically unresolved genera of various microlepidopteran families. In particular, the families Elachistidae, as well as Nepticulidae and Tortricidae, stand out with 7 and 7 unresolved species each, respectively. Only 3 species—*Crocallis* sp. (Geometridae), *Mythimna* sp. (Noctuidae), and *Nycteola* sp.—belong to Macrolepidoptera.

Some of these unresolved species have likely already been taxonomically described, but corresponding reference sequences are lacking in BOLD. The group of unassigned BINs certainly also includes undescribed species, some of which are already undergoing integrative taxonomic revision, such as *Dyseriocrania* sp. (Eriocraniidae) or *Exoteleia* sp. (Gelechiidae), a species that appears to remain undescribed despite sharing BIN with *E. dodecella*. Another example of complex taxonomic issues is the taxon previously referred to as *Eriocrania semipurpurella*, which likely comprises three species in Europe. While the recently described *E. marci* has also been confirmed in Austria (see below), the correct name of an additional genetic cluster remains unresolved and is therefore treated here as *Eriocrania* sp.

### 3.3. Unique Austrian BINs Attributed to a Linnaean Name—Genetic Endemism

The inventory currently comprises a total of 130 BINs known exclusively from Austria. 26 BINs show divergences of >4% from their nearest neighbour, whereas distances in 47 BINs are below 2% (Table S4).

In some cases, divergent BINs were detected within species that, based on current knowledge, are assigned to different BINs in neighboring faunas or elsewhere in Europe. This form of genetic endemism, which currently lacks formal taxonomic resolution, is of particular conservation relevance and warrants further detailed investigation.

**Figure 11:**
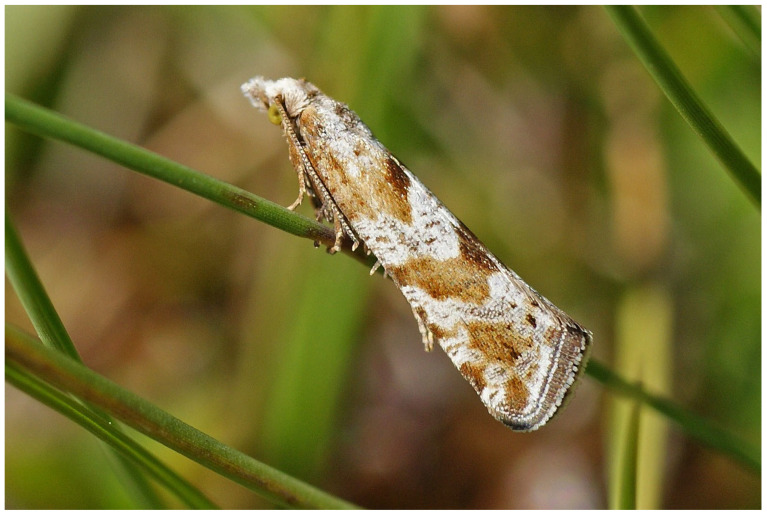
*Eriopsela klimeschi* is endemic to the Austrian Alps but shares its BIN with *E. quadrana.* Reproduced with permission from H. Deutsch.

In addition, regionally restricted genetic clusters include already known, morphologically well-established endemics of the country: *Stigmella geimontani*, *Lunakia alyssella*, *Dahlica reliqua*, *Dahlica styriaca*, *Dichrorampha dentivalva*, *Sphaleroptera dentana*, and *Kessleria hauderi*. Exceptionally, however, no unique BIN could be identified for morphologically unambiguous endemics such as *Eriopsela klimeschi* (Figure 11).

Among the endemic BINs, there are also 17 nominal species that exhibit conspicuously high intraspecific variability, being split into two to three BINs that appear to be restricted to Austria. These cases should likewise be examined for cryptic diversity in future integrative studies.

For a considerable proportion of the endemic BINs, this pattern likely reflects a sampling bias, as their distribution extends beyond Austria but genetic data from other regions are still lacking (e.g., *Agnathosia sandoeensis*, *Fuchsia luteella*, *Pseudophiaris sappadana*, or *Titanio normalis*.

**Figure 12:**
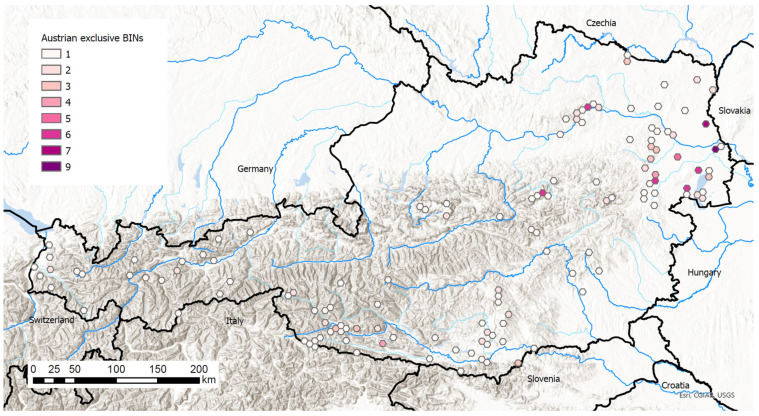
Geographical distribution of BINs known exclusively from Austria. Sampling sites that were close together were aggregated within hexagons with a diameter of 5 km. Map data source: ESRI, USGS, UBA.

Unique BINs known exclusively from Austria occurred in 133 hexagons and ranged from 1 to 9 per 5 km hexagon (Figure 12).

### 3.4. Faunistic Additions

Overall, since the beginning of our DNA barcoding activities, 90 genetically supported species have been recorded for the first time in Austria, together with a partly considerably larger number of new records for individual federal states [28,29,30,31,32,33]. Many of these new records were directly prompted by genetic surveys, whereas others were discovered using traditional morphological methods but have since been confirmed by DNA barcodes (Table S5).

In addition, there are 17 new records for Austria that remain unpublished to date:

Coleophoridae

*Coleophora graminicolella*: Burgenland, Neusiedl am See, Zitzmannsdorfer Wiesen, 4.viii.2021, leg. Huemer P.; Specimen IDs TLMF Lep 30952, TLMF Lep 31771; BIN BOLD:AAE1255.

*Coleophora potentillae*: Niederösterreich, Wollmansberg, Waschberg, 2.vi.2023, leg. Stark W.; Specimen ID BC_LSNOE_Lep_05469; BIN BOLD:ABY4881.

Cosmopterigidae

*Vulcaniella cognatella*: Burgenland, Breitenbrunn, NSG Thenauriegel, 8.vi.2021, leg. Huemer P.; Specimen ID TLMF Lep 31736; BIN BOLD:AEH3570.

Crambidae

*Achyra nudalis*: Kärnten, Arnoldstein, Riegersdorf, 23.viii.2019, leg. Vilgut M.; Specimen ID KLM Lep 15030; BIN BOLD:AAE2250.

Elachistidae

*Perittia obscurepunctella*: Kärnten, Virunum, Arena, Standort 1, 11.iv.2024, leg. Wieser C.; Specimen ID KLMLep16446; BIN BOLD:AAI4749.

Eriocraniidae

*Eriocrania marci*: locality and collecting data unknown, sequenced by Mutanen M.; Specimen ID MM17093; BIN BOLD:AAB3768.

Ethmiidae

*Ethmia iranella*: Burgenland, Jois, Hackelsberg, 11.v.2024, leg. Huemer P.; Specimen ID TLMF_Lep_43375; BIN BOLD:AGO7084.

Gelechiidae

*Acompsia baldizzonei*: Kärnten, Villach, 10.vi.2014, leg. Holzschuh C.; ditto, but 18.viii.2018, leg. Wieser C.; Specimen IDs KLM Lep 14350, KLM Lep 14410; BIN BOLD:ADR9697.

Heliozelidae

*Heliozela lithargyrellum*: Niederösterreich, Wollmansberg, Waschberg, 3.v.2022, leg. Stark W.; Wien, Penzing, Salzwiese, 5.v.2023, leg. Stark W.; Specimen IDs BC_LSNOE_Lep_04075, BC_LSNOE_Lep_04633; BIN BOLD:ADR1350.

Lyonetiidae

*Lyonetia padifoliella*: Nordtirol, Kauns NO, Trockenhang, 9.vii.2021, leg. Huemer P.; Specimen ID TLMF I.VAR. 00147; BIN BOLD:AAT9975.

Nepticulidae

*Stigmella szoecsiella*: Niederösterreich, Mannersdorf, alter Steinbruch, 23.viii.2024, leg. Stark W.; Niederösterreich, Neusiedl a. d. Zaya, Steinberg NO, 22.vii.2024, leg. Stark W.; Specimen IDs BC_LSNOE_Lep_05890, BC_LSNOE_Lep_05920, BC_LSNOE_Lep_05923, BC_LSNOE_Lep_05962; BIN BOLD:AAV8376.

Noctuidae

*Conisania cervina*: Niederösterreich, Mödling, 15.vi.1958, leg. Koch B.; Specimen ID BGE_ZSM_LEP_2720; BIN BOLD:AAR9913.

Pterophoridae

*Platyptilia picardi*: Niederösterreich. Lunz, Dürrenstein, Bärwiesboden, 18.vii.2016, leg. Stark W.; Specimen ID BC_LSNOE_Lep_01220; BIN BOLD:ABA2204.

*Stenoptilia aridus*: Vorarlberg, Zwischenwasser, Üble Schlucht, Eingangsbereich, 18.vi.2012, leg. Huemer P.; Vorarlberg, Lustenau, Schweizer Ried, AZE Häusle S, 25.vii.2012, leg. Huemer P.; Kärnten, Villach, Dobratsch, Nötsch, Hirschentumpf, 28.vii.2013, leg. Wieser, C.; Specimen IDs TLMF Lep 08096, TLMF Lep 08420, KLM Lep 01324; BIN BOLD:AAY8866.

Pyralidae

*Psorosa dahliella*: Burgenland, Jois, Hackelsberg, 11.v.2024, leg. P. Huemer; ditto, but 14.vii.2024; Specimen IDs TLMF_Lep_43376, TLMF_Lep_43572; BIN BOLD:ACA9753.

Tortricidae

*Eucosma suomiana*: Steiermark, Altaussee, Loserstrasse, 20.vi.2023, leg. Pöll N.; Specimen ID CCDB-39366-E05; BIN BOLD:AAF2288.

*Phalonidia udana*: Niederösterreich, Oberrohrbach, Goldenes Bründl, 3.vi.2016, leg. Stark W.; Niederösterreich, Lunz, Dürrenstein, Freiengraben, 19.vii.2022, leg. Stark W.; Specimen IDs BC_LSNOE_Lep_02083 BC_LSNOE_Lep_04316; BINs BOLD:ACF2757, BOLD:ACE8087.

Additional details on these species records are available in the public dataset on BOLD.

### 3.5. Taxonomic Insights from DNA Barcodes

Of particular interest for our study are numerous taxonomic discoveries. Fourteen species have been described since the last inventory, whose type material is based, at least in part, on Austrian material. DNA barcodes for all of these taxa have already been published in the original descriptions: *Agnoea jaeckhi*, *Agonopterix paraselini*, *Ancylis christiandiana*, *Batrachedra confusella*, *Callisto basistrigella*, *Coleophora avellanae*, *Coleophora cytisicolella*, *Coleophora ericarnella*, *Dichrorampha velata*, *Epermenia reinhardgaedikei*, *Nemophora scopolii*, *Phyllocnistis triandricola*, *Rhigognostis scharnikensis*, and *Symmoca schmidi* [6,34,35,36,37,38,39,40,41,42,43,44,45,46].

By contrast, records of the recently described *Gracilathetis kovacssandori* require re-evaluation [47]. DNA barcodes currently assigned to the sister species *G. lepigone* could belong to either of the two species. They correspond well with Finnish samples, a region where only the latter species occurs, raising the possibility of barcode sharing between the species or even taxonomic oversplitting.

In addition, several taxa that had been described earlier but were subsequently treated erroneously as synonyms were taxonomically revised. These results are based to a considerable extent on Austrian DNA barcodes and include the following species: *Antispila petryi*, *Caryocolum improvisella*, *Caulastrocecis cryptoxena*, *Cochylimorpha dorsimaculana*, *Chrysoclista gabretica*, *Coleophora paucinotella*, *Coleophora pannonicella*, *Epermenia plumbeella*, *Eupithecia conterminata*, *Gelechia obscuripennis* and *Hoplodrina alsinides* [48,49,50,51,52,53,54,55,56]).

*Agonopterix cluniana* has, however, been recognized as a synonym of *A. subtakamukui* based on new studies supported by DNA barcoding [57].

*Epiblema tussilaginana* is currently regarded as a valid species in Lepiforum [6]. However, the long-awaited revision has not yet been published [58].

Furthermore, *Eteobalea bernhardiella*, originally described from Austria as a subspecies of *E. tririvella*, is currently recognized as a valid species [59]. Our unpublished DNA barcode data provide additional support for its recognition as a distinct species.

Two further new species records in lepiforum, viz. *Stenoptilia asclepiadeae* and *Acrobasis fallouella* require careful reassessment and are omitted from Table S5.

## 4. Discussion

Despite ongoing efforts in DNA barcoding across Europe, comprehensive national barcode libraries for arthropods remain scarce. Finland is currently the only country with a fully documented national dataset [60]. Even in Germany, a leader in barcoding initiatives, many megadiverse insect orders still lack complete coverage, and the national Diptera library includes over 50% “dark taxa” [61]. Similarly, other species-rich orders, such as Hymenoptera, Coleoptera, and Lepidoptera, remain only partially represented [24,62,63]. In Austria, the Austrian Barcode of Life (ABOL) initiative has made notable progress since 2014, successfully processing smaller insect groups, although near-complete coverage of megadiverse orders has not yet been achieved [64,65].

In this context, the DNA barcode library presented here, covering 85% of Austrian Lepidoptera species, represents a significant advancement. It not only fills a critical gap in the genetic documentation of the Central European fauna but also provides a robust foundation for future biodiversity assessments, ecological studies, and conservation planning. These results underscore the value of targeted barcoding efforts for megadiverse insect orders and highlight the need to expand similar initiatives to other taxa and regions to achieve comprehensive genetic coverage.

The establishment of an Austrian DNA barcode library for Lepidoptera represents a major advance not only for taxonomy and faunistics but also for regional biodiversity conservation [66,67,68]. At the regional scale, DNA barcoding has enabled the detection of numerous new records for Austria and its federal provinces, indicating that current knowledge of species distributions is incomplete. At the same time, uneven spatial sampling highlights existing knowledge gaps and underscores the need for more systematic and geographically balanced survey efforts. The high-resolution genetic data now available provide a reliable basis for evidence-based biodiversity assessments, including the compilation and revision of regional Red Lists. Molecular data contribute to more precise evaluations of species’ conservation status and facilitate the identification of overlooked or misclassified taxa of potential concern.

However, despite the high coverage, several limitations should be considered. First, incomplete sampling, particularly in underrepresented regions, may bias estimates of species distributions and genetic diversity. Second, some taxa remain underrepresented or absent, especially rare, cryptic, or difficult-to-sample species. Third, reliance on a single genetic marker may limit resolution in cases of recent divergence, introgression, or incomplete lineage sorting.

Notwithstanding these limitations, the barcode dataset represents a valuable resource for continental and transcontinental genetic analyses and provides a basis for integrative species delimitation. Our results challenge the perception of a well-documented Central European fauna by revealing substantial hidden diversity, including cryptic and potentially undescribed species.

They also emphasize the importance of Austria for the conservation of distinct genetic diversity. In addition to well-documented endemics and potentially cryptic taxa, notable isolated genetic clusters occur within species currently regarded as unequivocal Linnaean taxa. A prominent example is *Lignyoptera fumidaria*, a species strictly protected under the EU Habitats Directive, which shows a divergence of approximately 3% from other populations.

This hidden diversity has direct implications for conservation, as it may lead to an underestimation of species richness and, consequently, misinformed conservation priorities. Accurate species delimitation is therefore essential for reliable assessments of species distributions, population trends, and threat status [69,70,71,72].

Overall, our study highlights the importance of integrative approaches that combine molecular and traditional methods in biodiversity research. Expanding DNA barcode reference libraries and integrating them into monitoring frameworks is essential for improving species documentation, informing conservation strategies, and addressing ongoing biodiversity loss.

## 5. Conclusions

This study demonstrates that the establishment of a national DNA barcode library for Lepidoptera in Austria provides substantial benefits for taxonomy, faunistics, and biodiversity conservation. The integration of molecular data has revealed previously undetected diversity, improved the accuracy of species identifications, and contributed to resolving long-standing taxonomic issues.

The application of DNA barcoding has also enhanced the quality and reliability of faunistic data, enabling more precise assessments of species distributions and facilitating the detection of new records at both the national and regional scales. These advances are particularly relevant for biodiversity monitoring and conservation planning, where robust data are essential for evidence-based decision-making.

However, remaining gaps in geographical coverage highlight the need for more systematic, spatially balanced sampling efforts in future studies. Expanding the DNA barcode reference library and further integrating molecular approaches into routine monitoring will be crucial for improving our understanding of species diversity and for supporting effective conservation strategies.

## Data Availability

All 23,463 COI sequences are available in the dataset BCAUT “Barcodes of Austrian Lepidoptera” https://doi.org/10.5883/DS-BCAUT (accessed on 7 April 2026).

## Supplementary Materials

The following supporting information can be downloaded at https://www.mdpi.com/article/10.3390/insects17050473/s1: Table S1: Checklist of Austrian Lepidoptera; Table S2: Species sharing BINs within Austria; Table S3: Taxonomically unresolved BINs; Table S4: BINs geographically restricted to Austria with Nearest Neighbor (NN) in BOLD; Table S5: Newly published species records for the fauna of Austria since the last checklist, supported by DNA barcodes. References [73,74,75,76,77,78,79,80,81,82,83,84,85,86,87,88,89,90,91,92,93,94,95] are cited in the Supplementary Materials.
